# Antiphospholipid Antibodies Overlapping in Isolated Neurological Syndrome and Multiple Sclerosis: Neurobiological Insights and Diagnostic Challenges

**DOI:** 10.3389/fncel.2019.00107

**Published:** 2019-03-19

**Authors:** Chiara D’Angelo, Oriol Franch, Lidia Fernández-Paredes, Celia Oreja-Guevara, María Núñez-Beltrán, Alejandra Comins-Boo, Marcella Reale, Silvia Sánchez-Ramón

**Affiliations:** ^1^Department of Clinical Immunology and IdISSC, Hospital Clínico San Carlos, Madrid, Spain; ^2^Department of Immunology, Ophthalmology and ENT, Complutense University School of Medicine, Madrid, Spain; ^3^Department of Medical, Oral and Biotechnological Sciences, University “G. d’Annunzio” Chieti-Pescara, Chieti, Italy; ^4^Department of Neurology, Hospital Ruber Internacional, Madrid, Spain; ^5^Department of Neurology, Hospital Clínico San Carlos, Madrid, Spain

**Keywords:** antiphospholipid syndrome, pathogenesis, MS-like syndrome, thrombosis, vasculopathy

## Abstract

Antiphospholipid syndrome (APS) is characterized by arterial and venous thrombosis, pregnancy morbidity and fetal loss caused by pathogenic autoantibodies directed against phospholipids (PL) and PL-cofactors. Isolated neurological APS may represent a significant diagnostic challenge, as epidemiological, clinical and neuroimaging features may overlap with those of multiple sclerosis (MS). In an open view, MS could be considered as an organ-specific anti-lipid (phospholipid and glycosphingolipid associated proteins) disease, in which autoreactive B cells and CD8+ T cells play a dominant role in its pathophysiology. In MS, diverse autoantibodies against the lipid-protein cofactors of the myelin sheath have been described, whose pathophysiologic role has not been fully elucidated. We carried out a review to select clinical studies addressing the prevalence of antiphospholipid (aPL) autoantibodies in the so-called MS-like syndrome. The reported prevalence ranged between 2% and 88%, particularly aCL and aβ2GPI, with predominant IgM isotype and suggesting worse MS prognosis. Secondarily, an updated summary of current knowledge on the pathophysiological mechanisms and events responsible for these conditions is presented. We draw attention to the clinical relevance of diagnosing isolated neurological APS. Prompt and accurate diagnosis and antiaggregant and anticoagulant treatment of APS could be vital to prevent or at least reduce APS-related morbidity and mortality.

## Current Challenges in The Diagnosis and Management of The Antiphospholipid Syndrome

The antiphospholipid syndrome (APS) is a systemic autoimmune disorder characterized by the presence of peripheral procoagulant autoantibodies together with the occurrence of recurrent thrombosis (venous, arterial or both) and/or pregnancy morbidity and fetal loss (Miyakis et al., 2006). The sole presence of autoantibodies does not always lead to thrombosis. APS can indeed be caused by a diverse array of antiphospholipid (aPL) antibodies that recognize cell surface proteins linked to phospholipids as “non self” within a pro-inflammatory context that has been described as a “second hit” (after infection or tissue damage). This combined effect would in turn activate the clotting cascade in a wide variety of mechanisms that lead to the development of thrombosis (Giannakopoulos and Krilis, 2013; Meroni et al., 2018).

The routine diagnostic aPL antibodies, used according to the 2006 Sydney revised APS classification criteria, are anticardiolipin (aCL), antiβ2-glycoprotein-I (aβ2GPI) and lupus anticoagulant (LA). The non-classic aPL antibodies include anti-phosphatidylserine (aPS), anti-phosphatidylserine-β2GPI (aPS-β2), anti-phosphatidylethanolamine (aPE), anti-prothrombin-prothrombin complex (aPT-PT), anti-phosphatidylserine-prothrombin complex (aPS-PT) and anti-annexin V (aAnV; Shoenfeld et al., 2008).

According to Sydney revision, classification of APS requires at least one clinical manifestation of vascular thrombosis or obstetrical events and the presence of at least two positive laboratory criteria (aCL IgG or IgM and/or aβ2GPI IgG or IgM at moderate titers and/or LA positivity) on two separate occasions at least 12 weeks apart (Miyakis et al., 2006). Persistence of positive aPL was introduced in order to differentiate the aPL antibodies appearing in the setting of infections or other unspecific conditions, in which aPL are transient and non-thrombogenic. Indeed, it is also well known that aPL antibodies fluctuate in blood, which hampers their interpretation (Donohoe et al., 2002; Fonseca and D’Cruz, 2008). To make the picture more complicated, besides the well-recognized obstetric and thrombotic hallmarks, APS can encompass an exceedingly variable clinical spectrum of multiorgan non-thrombotic manifestations, in the so called “extra-criteria” or “non-criteria” manifestations. Among these are the neurological manifestations, such as epilepsy, myelitis, chorea and migraine; hematological manifestations, such as thrombocytopenia and hemolytic anemia; livedo reticularis; pulmonary and osteoarticular manifestations; valvular heart disease; and nephropathy, to mention a few examples that cannot be exclusively explained by prothrombotic phenomena (Gómez-Puerta and Cervera, 2014; Negrini et al., 2017; Garcia and Erkan, 2018). In addition, the extra-criteria manifestations, as well as the classical ones, can occur in the setting of APS without fulfilling the serological criteria, as for instance with low titers’ aCL or aβ2GPI antibodies (Cobo-Soriano et al., 1999; Micheloud et al., 2005) or even in the absence of detectable aPL in the so-called “seronegative APS.”

APS may be diagnosed as an isolated disease (primary APS) or associated to other autoimmune disorders, mainly systemic lupus erythematous (SLE), rheumatoid arthritis, Sjögren syndrome, autoimmune thyroid disease, systemic sclerosis, systemic vasculitis, dermatopolymyositis, primary biliary cirrhosis and autoimmune hepatitis. It has been estimated that approximately 50% of patients who suffer from APS will develop SLE (Salmon et al., 2007). Nowadays, because of potentially recurrent thrombosis and the hypercoagulability scenario, there is consensus in treating APS patients with long-term oral anticoagulants and, in order to prevent obstetric manifestations, with a combination of low dose aspirin and low molecular weight heparin (LMWH;Empson et al., 2005).

There is a heated debate on the clinically significant titers of aPL antibodies. Investigators are strongly advised to classify patients as affected by APS, when more than one laboratory criterion is present alone or in combination. Specifically, LA presence in plasma; medium or high titer of IgG and/or IgM aCL antibody in serum or plasma (i.e., >40 GPL or MPL, or >the 99th percentile); IgG and/or IgM aβ2GPI antibody in serum or plasma (in titer >the 99th percentile; Miyakis et al., 2006). Growing evidences claim to consider the clinical impact of low level positive aPL antibodies and the necessity to set new cut-off levels, basically—though not exclusively—in obstetric APS (Devreese et al., 2010; Mekinian et al., 2012). The proposal to modify the APS classification criteria, mostly referring to laboratory requirements, is reinforced also by consideration that no differences were observed on obstetric complications, gestational period, arterial and/or venous thrombosis, when comparing pregnant women with aPL-related obstetric complications not fulfilling the Sydney criteria, with those fulfilling them (Arachchillage et al., 2015; Alijotas-Reig et al., 2018). Considering that also atypical (low or non-persistent), aPL antibodies presence may be associated with neurological disorders, such as transient ischemic attacks and migraine, but also epilepsy, transverse myelitis, multiple sclerosis (MS)-like syndrome, visual symptoms, dementia and chorea as well (Islam et al., 2016). The “rigid” adhesion to such criteria in clinical practice might exclude patients with “non-criteria” manifestations of APS in face of diagnostic uncertainty (Abreu et al., 2015; Aggarwal et al., 2015; Joseph and Habboush, 2018; Limper et al., 2018).

Whether anti-aggregant prophylaxis is needed in subjects with persistent positive aPL but without thrombosis history is still unclear. For the identification of the actual thrombotic risk, additional factors should be taken into consideration, such as hypertension, smoking, hypercholesterolemia, overweight or treatment with estrogens. Coexisting SLE and positivity to two or more aPL antibodies should be assessed too (Khamashta et al., 2016).

### Isolated Involvement of the Central Nervous System in the Antiphospholipid Syndrome

Neurological features, already predicted in the first description of APS in 1983 (Hughes, 1983), have not been included yet in the APS classification criteria. According to the 2006 APS classification criteria (Miyakis et al., 2006), only transient ischemic attack and stroke have been included as neurological manifestations. Nevertheless, a wide variety of neurological symptoms including cognitive dysfunction, psychosis, chorea and epilepsy cannot be solely explained by thrombotic events or hypercoagulability. These manifestations represent thus an important challenge in diagnostic practice, with a growing demand to recognize these “non-criteria” neurological manifestations in the classification of the disease (Abreu et al., 2015; Islam et al., 2016; Joseph and Habboush, 2018; Zhang and Pereira, 2018). A special case is the isolated central nervous system (CNS) involvement of APS, given the overlapping clinical and radiological features with MS (Achiron et al., 2004). In fact, isolated CNS APS usually occurs ranging from optical neuritis, chronic headache, migraine, cerebral ischemia, chorea, epilepsy, transverse myelopathy, to dementia and cognitive impairment (Hughes, 2003; Rodrigues et al., 2010). Most of these symptoms are usually referred as MS-like syndrome.

**Figure 1:**
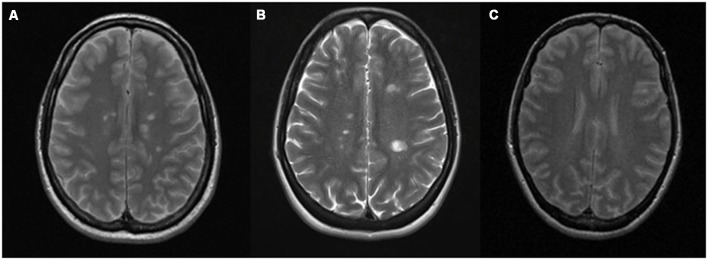
Conventional magnetic resonance imaging (MRI) showing similar demyelinating lesions in: **(A)** multiple sclerosis (MS) and **(B)** antiphospholipid syndrome (APS). **(A)** Gadolinium-enhanced T2-weighted sequence of a patient with MS showing multiple demyelinating lesions in the* supra* and infratentorial white matter, predominantly periventricular, without evidence of postcontrast enhancement. **(B)** T2-weighted sequence of a patient with APS that shows multiple focal demyelinating lesions in periventricular, juxtacortical posterior left parietal (by confluence of several lesions) white matter. that are hyperintense relative to the normal appearing brain tissue, indicating increased permeability of the blood–brain barrier. **(C)** Normal T2 MRI.

CNS APS and MS may be difficult to distinguish also from an immunological perspective, as the aPL antibody isotypes may involve IgG, IgM or IgA and therefore not always detected as the characteristic mirror pattern (positivity in serum and cerebrospinal fluid, CSF) or even of oligoclonal bands (OCB; predominant intrathecal antibody production; Cuadrado et al., 2000; Vilisaar et al., 2005). The two diseases resemble each other also for the epidemiological features of affected population, the relapsing-remitting course and for their appearance in neuroimaging (Figure 1). For both diseases, multifocal white matter lesions in magnetic resonance imaging (MRI) are the most common manifestation within the CNS (Chapman, 2004; Ferreira et al., 2005). In APS subjects, small strokes can occur in the white matter of brain and spinal cord, resulting in lesions that resemble the MS demyelinating plaques. The preferential localization is the subcortical area, and in a recent study, multiple subcortical and cortical infarcts with demyelination, involving both lobes of the brain, have been classified as characteristic MRI features for APS patients. White matter lesions were found in the periventricular area of the brain in almost the totality of the studied cases (Zhu et al., 2014).

Significant correlation between cognitive dysfunction and MRI lesions in primary APS patients has been reported, also in patients without CNS involvement (Tektonidou et al., 2006). In addition, vasculitis and inflammatory changes were also prevalent (Renaud et al., 2014).

Nowadays, the management and the treatment of APS patients with CNS involvement is still a matter of debate. There is good evidence of the benefit of anticoagulation in the typical thrombotic complications of APS, but there is still no consensus on the management with immunosuppression vs. anticoagulant therapy for non-thrombotic complications observed in MS-like syndrome. One might naturally wonder why, in the absence of any evident thrombotic injury on brain imaging, anticoagulant therapy should be used. In a case report by Zhang and Pereira (2018) an elderly woman with 6 months history of headache and intermittent choreiform movements of the face and arm, dramatically improved with warfarin therapy. Her blood tests showed positive LA, weakly positive aCL and negative aβ2GPI, with neither history of pregnancy loss or thrombosis. She received a trial of warfarin in the setting of probable APS, even if further investigation excluded potential secondary APS. After 2 weeks, her aberrant movements resolved as did her headaches, and since then she was symptom free for 18 months (Zhang and Pereira, 2018).

The evidence on how to manage movement disorders associated with APS are insufficient and no superiority of one drug to another has been demonstrated. In the reported case, a clear improvement of the patient’s life quality was achieved with anticoagulation, although the pathophysiology of movement disorders in APS, including chorea, as well as of other non-criteria neurological symptoms, remains poorly understood (Joseph and Habboush, 2018).

No standard treatment exists for non-thrombotic neurological manifestations of APS and available evidence mostly derives from retrospective non-randomized trials or case reports. In these studies anticoagulants have proven to be effective for the treatment of conditions that are not primarily thrombotic, such as migraine, transverse myelitis, and neuropsychiatric disturbances (Asherson et al., 2007; Roie et al., 2013).

According to the present state of knowledge, one might consider, under specific conditions, immunosuppressive treatment with corticosteroids, azathioprine and cyclophosphamide in addition to antithrombotic therapy, and combination with symptomatic management with neuroleptics (Cervera et al., 2014; Espinosa and Cervera, 2015; Yelnik et al., 2016). Rituximab, a monoclonal anti-CD20 antibody that depletes B cells, is currently used to treat various autoimmune diseases and hematological malignancies. Several case reports describe the use of rituximab in patients with APS, suggesting a beneficial role in the treatment and monitoring of refractory thrombocytopenia (Gamoudi et al., 2017) and recurrent thrombotic events in APS secondary to SLE (Emmi et al., 2017; Diószegi et al., 2018). The pilot therapeutic trial RITAPS that was designed to evaluate the efficacy and safety of rituximab in non-criteria APS manifestations (cognitive dysfunction, thrombocytopenia, cardiac valve disease, skin ulcers, nephropathy) did not show significant improvement of aPL profiles but a beneficial effect for a few of that conditions, given the small sample size. RITAPS was the first attempt to investigate immunosuppressive treatment in the management of cognitive dysfunction in aPL-positive patients without other systemic autoimmune diseases. The obtained findings indicated improvement in attention, visuomotor speed and flexibility (Erkan et al., 2013).

On the other hand, the rationale for the use of immunosuppressive and/or anticoagulant therapy could be given by the potential aPL-mediated damage considering each specific clinical case and clinical manifestations (Espinosa and Cervera, 2015).

## Antiphospholipid Syndrome Pathophysiology

**Figure 2:**
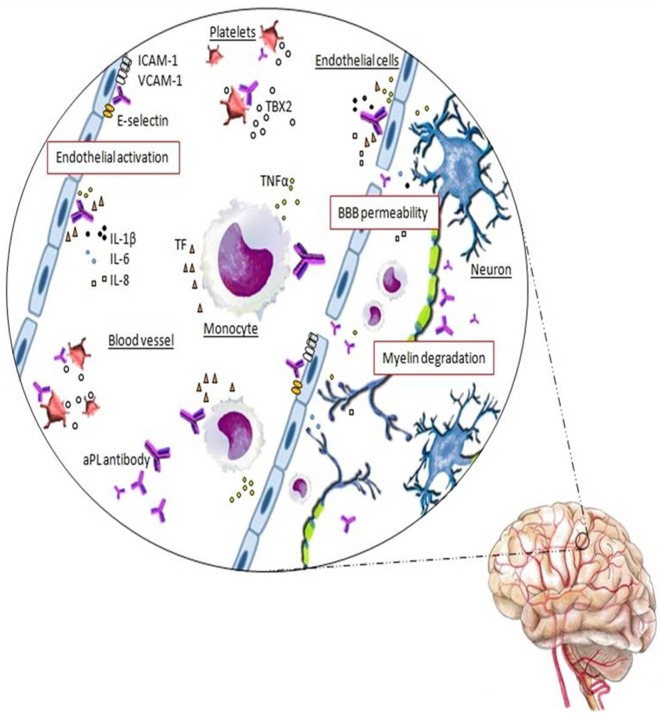
Schematic representation of the autoreactive response in APS in which the thrombus formation is mediated by the interaction of antiphospholipid (aPL) antibodies with the anticoagulant annexinV (AnV) on the surface of the endothelial cells, which are destroyed, accelerating coagulation cascade. Moreover, the binding of aPL antibodies promotes the endothelial cells activation determining an increased expression of adhesion molecules (ICAM-1, VCAM-1, E-selectin) and the release of tissue factor (TF), TNFα, IL-1β, IL-6 and IL-8 proinflammatory cytokines. aPL antibodies promote also platelets and monocytes activation. On the platelets membrane, anti-β2GP1 antibodies lead to platelets activation and thromboxane B2 (TBX2) release. In monocytes, aPL antibodies induce TF expression and TNFα production.The inflammatory condition determined by these events causes a vascular endothelium dysfunction with increased permeability of the blood-brain barrier (BBB). The neurons and myelin become now accessible to the aPL antibodies and their binding may trigger the myelin destruction and the neurodegeneration found in APS with MS-like syndrome.

aβ2GPI antibodies are central in many pathogenic APS mechanisms and, although the full pathogenesis of APS is not clear yet, the binding of these aPL antibodies to the antigens on the cell surface of platelets, monocytes, endothelial cells and trophoblasts, triggers intracellular signaling with subsequent activation and alteration of diverse cell functions. Monocytes and endothelial cells’ activation determine a pro-aggregation status due to up-regulated expression of adhesion molecules, such as E-selectin, and release of tissue factor (TF) and proinflammatory cytokines (Figure 2). Platelets’ activation and the subsequent release of thromboxane favor their aggregation. Cellular activation starts after the binding of the complex aβ2GPI antibody/β2GPI to the toll-like or annexin II receptors. Thrombosis at the fine vasculature of the target organ, such as retina or in the CNS, is thought to be more dependent from antibodies against the anticoagulant AnV. The resulting pathological and clinical events include inflammation and vasculitis, thrombus formation and arterial and/or venous vessel-occlusive disease (Pierangeli et al., 2006; Muscal and Brey, 2007; Merashli et al., 2015).

Although APS is considered an autoimmune condition mediated by specific production of autoantibodies and autoreactive T cells, innate immunity defects have been described as essential trigger factors within a multifactorial etiopathogenic scenario. As in most autoimmune diseases, genetically predisposed subjects, exposed to certain environmental agents could develop a specific immune response against self-proteins-binding phospholipids with a subsequent autoantibody production, together with the contribution of innate immunity mediators. Intrinsic alterations of the CNS myelin lipids or their cofactors (target tissue) could play a role in etiopathogenesis as well (Reale and Sanchez-Ramon, 2017). Coming up next, we summarize the events taking place in both innate and adaptive responses concerning APS.

### Innate Immune System in Antiphospholipid Syndrome Pathophysiology

APS is characterized by the presence of autoantibodies, but as previously mentioned, aPL antibodies is a necessary but not sufficient condition for the onset of the disease. Additional factors or a “second hit,” mediated by innate immunity, would be necessary to trigger the pathogenesis of the disease in the presence of autoantibodies, according to the current accepted theory (Pengo et al., 2011). In this context, certain environmental, proinflammatory or non-immunological procoagulant factors could induce the development of the disease, in genetically susceptible subjects (for instance, HLA-DR4 and HLA-Drw53 are risk factors; Matthey et al., 1989).

It must be also considered that, similarly to MS, several pathogens have been long described as potential triggers of the autoimmune response in APS. Molecular mimicry, with a modified-β2GPI from bacterial or viral structures may contribute to the development of the autoimmune response and the selection of autoantibodies (Cruz-Tapias et al., 2012).

aPL antibodies could be responsible of the specific activation of other innate immune cells and even non-immune cells. Presence of aβ2GPI antibodies induces up-regulation of TLR7 and TLR8 in plasmacytoid dendritic cells (pDCs; Prinz et al., 2011) and activates specific cells through the binding to relevant targets, such as TLR2, 4 and annexin A2 on monocytes and endothelial cells (Lambrianides et al., 2010; Satta et al., 2011; Allen et al., 2012); or ApoE receptor and glycoprotein Iba on platelets (Urbanus et al., 2008), promoting the development of a prothrombotic phenotype. It has indeed been demonstrated that knockout mice for these innate receptors, show a reduced thrombotic response following aPL antibodies administration (Pierangeli et al., 2007; Ramesh et al., 2011).

In addition, aPL antibodies directly promote up-regulation of the TF synthesis in monocytes (Sorice et al., 2007), neutrophils (Ritis et al., 2006) and endothelial cells (Kornberg et al., 2000). This procoagulant condition, not present under normal circumstances, plays an important role in contributing to the onset of thrombotic events in cancer, inflammation, angiogenesis and embryogenesis (Mackman, 2009).

aPL antibodies also interfere with the protein C function, especially through the competition for the phospholipid binding site, and therefore predispose to the development of venous thromboembolism (de-Groot et al., 1996).

Activation of the complement pathway is closely linked with thrombosis. Indeed, inhibition of the alternative complement pathway improves clinical outcomes reducing thrombosis risk (Chapin et al., 2016). aPL antibodies have shown to induce complement activation and to promote the upregulation of TF expression on neutrophils mediated by C5a (Ritis et al., 2006).

On the other hand, recent experimental studies on mice models suggest that natural killer T (NKT) cells play an important role in the regulation of aCL antibody production. NKT cells are characterized by their ability to recognize lipid antigens presented by CD1d molecules. Among a wide variety of self- and non-self lipids linked to CD1d in NKT cells, cardiolipin has been identified (Cox et al., 2009). NKT cells expansion was suggested to have a beneficial role in several autoimmune disorders through the release of immunomodulatory cytokines after antigen recognition (Godfrey and Kronenberg, 2004). This recognition of lipids, in the absence of exogenous antigens, is a hallmark of NKT cells and could account for activation and increased numbers of peripheral NKT cells described in several oncological and autoimmune diseases (Brigl et al., 2003; Darmoise et al., 2010). Although the exact mechanism is not well understood, it has been demonstrated that NKT cells can regulate the activation of autoreactive B cells in a CD1d-dependent manner (Wermeling et al., 2010; Yang et al., 2011). In contrast, absence or reduction of NKT cells or of CD1d-expression on B cells have been related with an increased autoreactive B cells activation and higher aPL release, which raised the hypothesis that aPL antibodies could result from imbalanced NKT population. These data point to NKT cells as an intriguing therapeutic strategy to establish tolerance, a key element for ameliorating autoimmune diseases (Wermeling et al., 2010).

### Adaptive Immune System in APS Pathophysiology

Undoubtedly, aPL auto-antibodies production by autoreactive B cells is the key feature of APS, although little is known about their emergence, their overactivation, regulation and about all their pathological effects. In line with the second hit theory, molecular mimicry has been proposed as a potential underlying mechanism for aPL antibodies production (Blank et al., 2002; Gharavi et al., 2002, 2003; Shoenfeld et al., 2006; Martin et al., 2011). In humans, many viral or bacterial infections have been associated with the production of IgM and IgG aPL, which can be persistent in time. As β2GPI has a similar aminoacidic sequence to that of several bacterial and viral components, it has been postulated that subjects with a certain genetic background may produce cross-reactive antibodies (Abdel-Wahab et al., 2016). For many years, aPL antibodies have been considered natural antibodies because of their polyspecific repertoire and other similar characteristics to those produced by B1 cells (Youinou and Renaudineau, 2004; Merrill, 2006). Conversely, aPL antibodies have been shown to be mainly of IgG and IgA isotype (Fanopoulos et al., 1998), hence probably their secretion needs to be T-cell dependent. In fact, a specific T cell-response against β2GPI in APS patients has been reported by several investigators (Kuwana, 2003; Yamaguchi et al., 2007). An increase in IL-17/IL-23 indicating a Th17 response has been described in APS as well (Meroni et al., 2011; Popovic-Kuzmanovic et al., 2013; Jakiela et al., 2016). Although there is no definitive evidence to prove these concepts, it has been demonstrated that antigen driven maturation increases the pathogenic potential of aPL antibodies, although it is not an indispensable prerequisite for pathogenicity (Lieby et al., 2004). In fact, aPL antibodies production induced by infection has not shown to be pathogenic, even if significantly higher number of infected patients with aPL antibodies titer develop thrombotic events. Further, serum collected from healthy individuals can present these autoantibodies too (Uthman et al., 1999; Justo et al., 2011; Nakayama et al., 2014; Abdel-Wahab et al., 2016). To date, it is universally accepted that aPL antibodies cause the typical clinical manifestations of APS, but the causal relationship with the thrombotic events remains speculative.

Besides the diverse effects previously described on innate immune cells, aPL antibodies appear to interfere with the natural antithrombotic processes, by recognition and binding to β2GPI, expressed on the surface membranes of several cell types involved in the coagulation cascade, as well as decidual stromal cells, throphoblast cells, glial cells and neurons (Borghi et al., 2007; Lavazza et al., 2007). The former have been reported to induce defective *in vitro* trophoblast proliferation and differentiation as well as increased apoptosis (Di Simone et al., 2007).

aPL antibodies’ prothrombotic effects have also been observed in the CNS. Intrathecal synthesis of aPL antibodies or the disruption of the blood-brain barrier (BBB) could allow aPL autoantibodies to enter the CNS (Martínez-Cordero et al., 1997). Prothrombotic events could be triggered by direct interaction between aPL antibodies and CNS-resident cells. aPL antibodies have been related to astrocyte proliferation and nonspecific permeabilization and depolarization of synaptoneurosomes *in vitro* (Chapman et al., 1999).

The most characteristic features of APS affecting CNS, have been reported to be the connective dysfunction disorder and demyelinization (Espinosa and Cervera, 2008), but the specific role of aPL antibodies in non-thrombotic CNS manifestations of APS or transverse myelitis, remains to be established. Even though studies on small cohorts of patients have shown specific non-thrombogenic effects of aPL antibodies on CNS, with both vascular and non-vascular damage involved in the neurological manifestations of the disease. Data suggest a direct binding of aPL antibodies within the CNS, inducing activation of astrocytes, neurons and brain endotheliocytes (Figure 2). However, the binding of aPL antibodies to the membrane phospholipids within the brain has not been extensively studied. Caronti et al. (1998a,b) demonstrated that the neuronal damage might occur by a direct interaction of aPL antibodies with neurons or by functional impairment after their interaction with astrocytes, endothelial cell activation and adherence to CNS cells. These authors showed by indirect immunofluorescence that aβ2GPI antibodies purified from the serum of a patient with SLE/APS specifically bound CNS cells, in particular astrocytes and neurons in culture and in histological sections of human and monkey brain, and to cerebral vascular endothelium.

aCL antibodies’ binding was demonstrated also in a cell line of rat astrocytes. In these cells, aCL antibodies exerted an inhibitory effect by decreasing the cells’ viability and by depolarizing the cell membrane, impairing the signal transduction (Sun et al., 1992).

Chapman et al. (1999) in 1999 speculated about the potential target site of aPL antibodies at the neuronal synapses. They examined aPL antibodies effects on the plasma membrane function of rat synaptoneurosomes with IgG aPL purified from APS patients’ sera. The study was a proof-of-concept describing the functional interaction of IgG purified aPL antibodies from APS subjects with neuronal cell membranes, showing increased depolarization and permeabilization of synaptoneurosomes.

Moreover, it should be considered that by targeting antigens at the BBB and compromising its integrity, aPL antibodies in APS patients gain access to the CNS (Figure 2). Further, supporting the hypothesis of a direct role of these autoantibodies in the pathogenesis of neurological manifestations, *in vivo* mice behavioral tests were performed by Shoenfeld et al. (2003). After direct injection of IgG aPL purified from APS patients into mice brain, worst performances were demonstrated in the animals.

Despite these studies were conducted long-time ago, whether neurological hyperexcitatory manifestations, such as epilepsy and chorea could result from aPL-induced synaptic depolarization or whether the neuronal depolarization might explain the dementia and cognitive dysfunction found in APS, remains unknown.

The CNS involvement in APS might be additionally mediated by a direct neurotoxic effect of aPL antibodies that leads to impaired basal ganglion cell function with the development of neuroinflammation. Possibly, the aPL antibody bond to the cerebral endothelium could also cause endothelial dysfunction and lead to microthrombosis and inflammation of the blood vessels as well. This hypothesis might provide an explanation of why anticoagulation or immunosuppression therapy can represent at present an effective treatment in these aPL positive patients with neurological features. The above-mentioned mechanisms may be relevant not only in APS but in aPL positive MS patients, given the parallel mechanisms that cross between the two conditions.

In addition, a hypothesis proposes that the already described MS-like manifestations in APS patients could depend on the direct reactivity of aPL antibodies and myelin antigens by “molecular mimicry” and cross-reacting with myelin, myelin-related proteins and the cerebral phospholipids cephalin and sphingomyelin (Figure 2; Rombos et al., 1990; Karussis et al., 1998; Cikes et al., 2008). MRI studies of APS patients frequently show multiple T2-hyperintense brain lesions. Thus, typical demyelinating lesions of MS, transverse myelitis and optic neuritis, may also be present in the pathological spectrum of APS (Cikes et al., 2008). Moreover, preliminary data showed that the molecular mimicry of aPL-target antigens with myelin, myelin-related proteins and brain phospholipids may lead to cross-reactivity and predispose to a prothrombotic state (Koudriavtseva et al., 2014; Uthman et al., 2015).

## Prevalence of Non Organ-Specific Antiphospholipid Antibodies in Multiple Sclerosis

MS is an autoimmune mediated inflammatory and neurodegenerative disease characterized by multifocal areas of inflammation, due to autoreactive T and B lymphocytes and macrophage infiltrations, which cause demyelination, axonal damage with neuronal loss and gliosis, within both the white and gray matter of the CNS (Machado-Santos et al., 2018). These events lead to the formation of lesions, called plaques, which interfere with nerve impulses’ transmission. The nervous transmission alteration accounts for the clinical MS features, such as autonomic and sensory defects, loss or reduction of motor functions and paralysis, fatigue, speech disorders, ataxia, difficulties in concentrating/thinking, learning and memory impairment and psychological problems (Compston and Coles, 2008).

The well-known heterogeneity of MS reflects a myriad of pathogenic mechanisms contributing to the disease. MS pathogenesis is mainly considered to be mediated by Th1 and Th17 autoreactive T cells infiltrating the CNS, which initiate an inflammatory cascade causing destruction of myelin sheath, oligodendrocyte and microglia damage, and finally axonal and neuronal destruction. Lipid-reactive NKT cells have been suggested to primarily drive Th1 and Th17 responses, after activation between certain myelin glycosphingolipids (particularly the derivatives of galactosylceramides) and CD1d glycoprotein (De Libero et al., 2007; Blewett, 2008; Hogan et al., 2013). Phospholipids and glycosphingolipids are the major components of CNS myelin sheath that, under certain circumstances, could become immunogenic and trigger autoimmune responses (Reale and Sanchez-Ramon, 2017). Moreover, invariant NKT (iNKT) cells seem to have a dual proinflammatory vs. counter-regulatory role in MS responses (Podbielska et al., 2018).

To date, B cells and humoral response in MS pathogenesis have reached more importance based on clonally expanded memory B cells and anti-myelin-specific lipids autoantibodies detected in MS patients, as indicated by the diagnostic use of OCB. The presence of the characteristic OCBs in the CSF is one of the main hallmarks of MS although not specific, together with antibody deposition, complement activation and demyelination.

**Table 1:** Summary of relevant studies on the prevalence of aPL in MS patients.

Study	Population	aPL antibodies positivity
Filippidou et al. (2016)	127 MS (Serum); 88 RR, 11 PP, 28 SP	RR: aCL: 10.2% IgM, 18.2% IgG PP: aCL: 36.4% IgM, 18.2% IgG SP: aCL: 35.7% IgM, 32.1% IgG
Mandoj et al. (2015)	100 MS (Serum); 58 REM, 26 REL, 16 SP	REM: aCL: 1.7% IgM, 1.7% IgG, aβ2GPI: 1.7% IgM, aPT: 3.4% IgM, 5.2% IgG, aAnV: 1.7% IgM, 6.9% IgG REL: aCL: 7.7% IgM, 11.5% IgG, aβ2GPI: 26.9% IgM, aPT: 15.4% IgM, 19.2%IgG, aAnV: 3.8% IgM,15.4% IgG SP: aCL: 6.3% IgM, 6.3% IgG, aβ2GPI: 6.3% IgM, aPT: 6.3% IgM, aAnV: 6.3% IgM, 18.8% IgG
Shor et al. (2015)	98 MS (Serum)	aPS-β2: 14.6% IgM, 22.4% IgG, aPT: 46.9% IgM, aPT-PT: 71.4% IgG
Koudriavtseva et al. (2014)	100 MS (Serum)	aCL: 4% IgM, 5% IgG, aβ2GPI: 9% IgM, aPT: 7% IgM, 8% IgG, aAnV: 3% IgM, 11% IgG
Szmyrka-Kaczmarek et al. (2012)	85 MS (Serum)	aβ2GPI: 20% IgM, aCL: 4.7% IgM, 1% IgG
Stosic et al. (2010)	49 MS (Serum)	aCL: 18.4%, aβ2GPI: 10.2%, aPS: 18.4%, aPE: 32.6%
Garg et al. (2007)	111 MS, 27 CIS (Serum)	MS: aCL: 6%, aβ2GPI: 2%, CIS: aβ2GPI: 4%
Bidot et al. (2007)	24 RRMS (Serum); 7 REM, 17 REL	REM: aβ2GPI: 28% IgM, aCL: 28% IgM, aPS: 14% IgM, aPE: 28% IgM, aPC: 14% IgM REL: aβ2GPI: 82% IgM, aCL: 82% IgM, aFVIIa: 59% IgM, aPS: 71% IgM, aPE: 82% IgM, aPC: 76% IgM
Roussel et al. (2000)	89 MS (Serum)	aCL: 4.5% IgM, 16.9% IgG aβ2GPI: 13.5% IgM, 2.2% IgG
Karussis et al. (1998)	170 MS (Serum); 100 atypical, 70 classical	aCL: 27% atypical MS aCL: 5.7% classical MS
Sugiyama and Yamamoto (1996)	32 MS (Serum)	aCL: 44% IgM, 9% IgG
Marchiori et al. (1990)	33 MS (Serum, CSF)	aCL: 46.2% IgG (CSF)

*MS, multiple sclerosis; RR, relapsing remitting; PP, primary progressive; SP, secondary progressive; REM, remission; REL, relaps; CIS, clinically isolated syndrome; CSF, cerebrospinal fluid; Acl, anti-cardiolipin; aβ2GPI, anti-β2glycoprotein I; aAnV, anti-annexin V; aPS-β2, anti-phosphatidylserine-β2GPI complex; aPT, anti-prothrombin; aPT-PT, anti-prothrombin complex; aPE, anti-phosphatidylethanolamine; aPS, anti-phosphatidylserine; aPC, anti-phosphatidylcholine; aFVIIa, anti-factor VII activated*.

The first and the most common detected autoantibodies in MS recognize the complexes of membrane proteins assembled to myelin lipids, like the transmembrane proteolipid protein (PLP), the extrinsic myelin basic protein (MBP), the myelin oligodendrocyte glycoprotein (MOG) and the myelin associated glycoprotein (MAG; Kanter et al., 2006; Podbielska and Hogan, 2009). Antibodies directed against glycolipids like ganglioside have been reported too (Stevens et al., 1992). Specific autoreactive T-cells and autoantibodies’ reactivity directed against lipids such as sulfatide, phosphatidylcholine and sphingomyelin, and also against lipids that are altered by oxidative processes within the brain tissue of MS patients, including cholesterol, phosphatidylcholine, phosphatidyl ethanolamine and lysophosphatidyl ethanolamine have been described (Kanter et al., 2006; Fraussen et al., 2014). These antigenic stimuli from oxidized lipids, may overlap with those involved in the pathophysiology of APS. In MS, it has been evoked the role of capillary and venous hemorrhages that result in extracellular release of hemoglobin and reactive molecules that could induce local oxidative stress, inflammation and tissue damage. In fact, oxidized extracellular hemoglobin cause direct oxidative damage to myelin components, specifically to MBP (Bamm et al., 2017). In this context, vascular pathology could exert a primary event in the induction of a subsequent immunogenic response in MS. In an open view, MS could be considered as an organ-specific aPL disease, in which autoreactive B cells and CD8^+^ T-cells play a major role in its pathophysiology (Machado-Santos et al., 2018).

The use of MRI and the paramagnetic element gadolinium (Gd) in MS is useful to detect CNS plaques with active inflammation and lesion burden. Gd can only cross the damaged BBB at sites of tissue destruction or inflammation. However, the correlation between Gd-enhancing lesions and cognitive deficit is weak or absent in MS (Rocca et al., 2015).

Different MS clinical forms have been described, including relapsing remitting (RRMS), the most common subtype (approximately affects 87% of patients), primary progressive (PPMS), secondary progressive (SPMS) and progressive relapsing (PRMS). RRMS is characterized by acute inflammatory attacks, known as exacerbations or relapses, followed by periods of remission between relapses. During every attack, destruction of myelin and nerve fibers occur and accumulate in the long-term. Nearly 65% of patients with RRMS will subsequently develop SPMS, which is considered the second phase of the disease (Ghasemi et al., 2017).

MS different clinical forms are considered to translate into variable pathophysiological pathways, determining different patients’ prognosis and treatment decisions. To-date, there is no cure for primary progressive MS, while there are diverse disease-modifying treatments (DMTs) for RRMS and SPMS in current use. The most common strategy of MS management is the “escalation therapy” beginning with interferon-β, glatiramer acetate, and corticosteroids for acute relapses. In patients with demonstrated moderate-to-high-disease activity, early initiation of “high-efficacy” DMTs, such as fingolimod, monoclonal antibodies such as natalizumab and alemtuzumab and new generation monoclonal anti-CD20 antibodies such as ocrelizumab may help to better control the disease (Merkel et al., 2017). These immunomodulatory and anti-inflammatory treatments can partially diminish disease progression and alleviate MS symptoms, by exerting inhibition of immune cell activation and migration through the BBB, lymphocyte proliferation and macrophage-mediated myelin degradation and blocking secretion of proinflammatory cytokines, among other effects (Torkildsen et al., 2016; Ghasemi et al., 2017; Nandoskar et al., 2017).

The hypothesis that aPL antibodies may be involved in the pathogenesis of MS, and the potential association between their presence and specific MS clinical subtypes or clinical phase is not recent (Marchiori et al., 1990; Sugiyama and Yamamoto, 1996; Karussis et al., 1998; Roussel et al., 2000; Horstman et al., 2009). Interestingly, PL/PL-linked cofactor antigens are clinically relevant in MS. We carried out a review in several steps: (1) articles were identified and revised by a computer assisted search of published reports (PubMed, US National Library of Medicine, National Institutes of Health) to locate all cases of MS in which aPL antibodies were analyzed and their positivity was reported. Bibliographies of each article were also scanned for references not identified in the initial search. Only cases with well documented clinical summaries and relevant information were included in the review. Data from these articles were summarized using a standardized data form, including population size, diagnosis and MS clinical phase, sample type, and the percentage of positivity for the specific antibody. According to our review, non organ-specific aPL autoantibodies, particularly aCL and aβ2GPI, that could account for APS patients with MS-like syndrome, occur within a range between 2% and 88% according to the different studies, with predominant IgM over IgG isotype, both in serum and CSF (Table 1). Due to the conflicting published levels, the exact prevalence, pathogenic role and clinical significance of aPL antibodies in MS remain still unclear and highly debated.

In 2007, two studies found higher aPL antibodies titer in later MS phases, aCL and PE were more common in SPMS when compared to RRMS (Bidot et al., 2007; Garg et al., 2007). By studying MS during the exacerbation phase, Bidot et al. (2007) found a higher titer of IgM aβ2GPI and aCL in 82% of patients with respect to those in remission phase (28%).

Koudriavtseva et al. (2014) found significantly higher frequency of aPL antibodies in MS subjects (32%) compared to healthy controls (7%), with a higher titer in RRMS patients (53.8%). Increased prevalence of IgM β2GPI in MS patients (20%) vs. controls (3.3%), was detected also by Szmyrka-Kaczmarek et al. (2012) of whom 33% were in SPMS patients and 21% in RRMS patients.

More recent studies have focused as well on aPL autoantibodies incidence in MS patients in different clinical phases of the disease, showing at least one IgM aPL or IgG isotype elevated in MS subjects, in RRMS or SPMS phases (Mandoj et al., 2015; Shor et al., 2015; Filippidou et al., 2016).

Positive aPL in the setting of local inflammatory status may induce any of the diverse pathogenetic mechanisms previously mentioned. aPL antibodies may account for antibody-mediated complement deposition in a portion of MS demyelinating lesions and might underline a proportion of approximately 30% of MS patients in which hypoxia-like pathological findings are found (Lucchinetti et al., 2000). Presence of aPL antibodies in SPMS or at later stages of MS might be indicative of a more chronic and worst course of CNS injury. Therefore, positive aPL antibodies in MS might associate to MRI lesions, as their detection could reveal more severe lesions in MS patients with aPL antibodies positivity with respect to those with lower or absent aPL titers (Stosic et al., 2010). However, these correlations still remain to be further investigated.

## Overlap Between Antiphospholipid Syndrome and Multiple Sclerosis: Clinical and Prognostic Implications

Nowadays, there are no definite diagnostic tools for distinguishing atypical MS and neurological APS cases, and the occurrence of positive aPL autoantibodies in patients with MS, in the absence of systemic manifestations of autoimmune disease or APS, is of particular concern. Therefore, it seems probable that a small percentage of patients diagnosed with MS, do in fact have a primary neurological APS, a condition with an entirely different pharmacological treatment and that would condition prognosis too. APS misdiagnosis as MS makes a crucial point for the therapeutic approach, given the increased prothrombotic risk (Fernández-Fernández et al., 2006; Donnan and McDonald, 2009; Ahbeddou et al., 2012). Finally, the coexistence of both autoimmune diseases, like APS secondary to MS, could also be possible.

An acute clinical isolated neurological syndrome (CIS) poses the biggest diagnostic challenge, since it is the most common onset of MS, but can also be the only feature or the first manifestation in APS before the occurrence of other features, such as thrombosis or miscarriages (Ferreira et al., 2005). An article published in The Times newspaper reported the results of a survey of the London Lupus Centre suggesting that at least 5% of MS patients were misdiagnosed and suffer from APS instead of MS (Rose, 2006).

APS patients have a generally good clinical outcome under anticoagulant treatment; manifestations such as headache and memory loss often improve drastically, with no further neurological events, when they are properly anticoagulated (Cuadrado et al., 2000).

For the above reasons, APS is recognized as a severe but potentially treatable condition, considering also the neurological complications. However, no standard treatment is available yet for the aPL-associated neurologic manifestations not included in the APS classification criteria, and the effects of immunosuppressive and anti-inflammatory agents, usually used in MS, is unknown in these patients (Espinosa and Cervera, 2015). A careful and correct diagnosis could be vital to avoid or at least reduce APS-related morbidity and mortality.

Despite MS is an incurable neuroinflammatory and neurodegenerative disease, a prompt and adequate treatment, focused on control of MS relapses, partially ameliorates accumulation of physical and neurological disability in the long-term. In fact, relapses timing is unpredictable but during every attack, destruction of myelin occurs and destroyed axonal fibers accumulate in CNS with worsening of patient’s clinical condition. The early initiation of DMTs leads to improved stability control of MS disease, when compared to delayed therapy onset (Kavaliunas et al., 2017; Merkel et al., 2017). The presence of aPL antibodies in MS may herald a misdiagnosis of APS or the coexistence with APS, implying the establishment of anticoagulant therapy and the improvement of the prognosis for the individual patient.

## Conclusion

APS and MS may be both considered as anti-lipid autoimmune diseases with specific pathophysiological mechanisms and events, given the direct role of antiphospholipid in the coagulation cascade, which can cross in the individual patient. Since the time they have been defined, clinical findings cannot clearly distinguish between atypical MS and neurological APS; basic and clinical research is still needed to reduce the misdiagnosis in these difficult cases. In these patients, to date, an accurate diagnosis may only emerge after long-term follow-up. Primary or secondary APS has to be considered an essential differential diagnosis from MS because prompt and correct treatment can improve quality of life and may also reduce morbidity and mortality in the affected patients. Understanding the possible associations between aPL antibodies and non-stroke neurological disabilities warrants further research. The knowledge of new pathogenic mechanisms of aPL might identify novel therapeutic targets and therefore improve the clinical management of atypical APS and aPL-positive MS patients.

## Author Contributions

CDA and SS-R designed the work and wrote the first draft of the article. LF-P and MR substantially contributed to writing. All authors have revised and approved the manuscript.

## Conflict of Interest Statement

The authors declare that the research was conducted in the absence of any commercial or financial relationships that could be construed as a potential conflict of interest.
